# Demographic Divergence History of Pied Flycatcher and Collared Flycatcher Inferred from Whole-Genome Re-sequencing Data

**DOI:** 10.1371/journal.pgen.1003942

**Published:** 2013-11-07

**Authors:** Krystyna Nadachowska-Brzyska, Reto Burri, Pall I. Olason, Takeshi Kawakami, Linnéa Smeds, Hans Ellegren

**Affiliations:** Department of Evolutionary Biology, Evolutionary Biology Centre, Uppsala University, Uppsala, Sweden; University of Wisconsin–Madison, United States of America

## Abstract

Profound knowledge of demographic history is a prerequisite for the understanding and inference of processes involved in the evolution of population differentiation and speciation. Together with new coalescent-based methods, the recent availability of genome-wide data enables investigation of differentiation and divergence processes at unprecedented depth. We combined two powerful approaches, full Approximate Bayesian Computation analysis (ABC) and pairwise sequentially Markovian coalescent modeling (PSMC), to reconstruct the demographic history of the split between two avian speciation model species, the pied flycatcher and collared flycatcher. Using whole-genome re-sequencing data from 20 individuals, we investigated 15 demographic models including different levels and patterns of gene flow, and changes in effective population size over time. ABC provided high support for recent (mode 0.3 my, range <0.7 my) species divergence, declines in effective population size of both species since their initial divergence, and unidirectional recent gene flow from pied flycatcher into collared flycatcher. The estimated divergence time and population size changes, supported by PSMC results, suggest that the ancestral species persisted through one of the glacial periods of middle Pleistocene and then split into two large populations that first increased in size before going through severe bottlenecks and expanding into their current ranges. Secondary contact appears to have been established after the last glacial maximum. The severity of the bottlenecks at the last glacial maximum is indicated by the discrepancy between current effective population sizes (20,000–80,000) and census sizes (5–50 million birds) of the two species. The recent divergence time challenges the supposition that avian speciation is a relatively slow process with extended times for intrinsic postzygotic reproductive barriers to evolve. Our study emphasizes the importance of using genome-wide data to unravel tangled demographic histories. Moreover, it constitutes one of the first examples of the inference of divergence history from genome-wide data in non-model species.

## Author Summary

Demographic processes leave specific and detectable signatures within species genomes. Analysis of patterns of variation within and between closely related species can be used to unravel their divergence history and is crucial for understanding evolutionary processes such as speciation. We applied a set of novel population-genomic tools to investigate patterns of natural variation and infer demographic history of two avian speciation model species: pied flycatcher and collared flycatcher. The analysis supported a scenario consistent with allopatric speciation with recent, postglacial secondary contact. Most likely the ancestral species persisted through one of the glacial periods of the middle Pleistocene and then split into two large descendent populations that appear to have increased in size before experiencing severe bottlenecks during expansion into their current ranges. The two species established secondary contact after the last glacial maximum. This resulted in unidirectional gene flow from pied flycatcher to collared flycatcher. The results are consistent with a scenario where pied flycatcher recolonized northern Europe more rapidly than collared flycatcher. Our study increases the knowledge about the dynamics of the speciation process and constitutes one of the first examples of the inference of complex demographic history using information from genome-wide data in non-model species.

## Introduction

Considerable attention is currently paid to the role of gene flow during speciation [1]–[4]. In the presence of gene flow, strong ecology-driven divergent selection is an important initial prerequisite for the evolution of reproductive isolation. In allopatric speciation, the initial stages of speciation can be facilitated by genetic drift and local adaptation in geographic separation without being countered by gene flow. Still, gene flow may occur in secondary contact via introgressive hybridization and in some cases boundaries may then collapse [5]–[10]. Irrespective of the role of selection and geographic separation, the evolution and maintenance of reproductive isolation in the face of gene flow is expected to generate a genomic mosaic in which regions permeable to gene flow are less differentiated than regions resistant to introgression [11]. Such mosaics are characterized by the presence of ‘genomic islands of speciation’ [12], [13], genome regions which may harbor loci under divergent selection and potentially underlie reproductive incompatibility. However, what processes contribute to these patterns is still a matter of debate. Under the model of divergence hitchhiking [4], [13], [14], such regions can be extensive, with reduced genetic exchange over several megabases (Mb) of linked sequence. However, it is the remaining regions of the genome that harbor information about patterns of gene flow and other demographic processes that, apart from different types of selection, influence species differentiation. Moreover, in order to correctly infer the evolutionary and population processes causing localized elevated differentiation, it is imperative that background levels of gene flow are well characterized.

A wide range of approaches have been developed to estimate demographic history and/or the role of gene flow during (and after) speciation. Particularly relevant recent developments include numerous coalescent-based methods (e.g. [15]–[18]) that estimate ancestral population sizes, historical gene flow, and divergence times. The coalescent offers a powerful theoretical framework for such analyses [19]–[21] and coalescence modeling is increasingly used in the context of speciation research [22]–[25]. The isolation-with-migration (IM) model of Hey and Nielsen [24] has been successfully applied in the past to distinguish ancestral polymorphism from introgression and to estimate divergence history and the role of gene flow during speciation in many species (reviewed in [26]). However, it exclusively considers demographic scenarios with constant migration rates between species, and thus offers no means to investigate more complex patterns of gene flow over time. Moreover, it is computationally demanding (due to likelihood function evaluation) and its use is limited to rather small datasets [26]–[31]. The Approximate Bayesian Computation (ABC; [32]) approach bypasses exact likelihood calculation by using summary statistics to characterize patterns of variation observed in the data. The approach is also very flexible in defining demographic models used to infer demographic parameters [33]–[37]. Since their first implementation in population genetics, ABC methods have been constantly developed and improved [18], resulting in an increasing number of studies inferring demography within an ABC framework [33], [34], [37], [41]–[50].

Though coalescent modeling can handle genome-wide data, its application for genome-wide demographic inference has so far been restricted by the limited access to whole-genome sequence data. Notable exceptions include studies of the demographic history of humans [41], [51]–[53], other primates [17], [42], [54], [55], and *Drosophila melanogaster* [47]. With the emergence of the field of speciation genomics and the foreseeable increase in the number of non-model genomes sequenced [56], an increase in the number of studies inferring population history of important study organisms from genome-wide data is also to be expected. Here we present one of the first examples in this direction.

**Figure 1:**
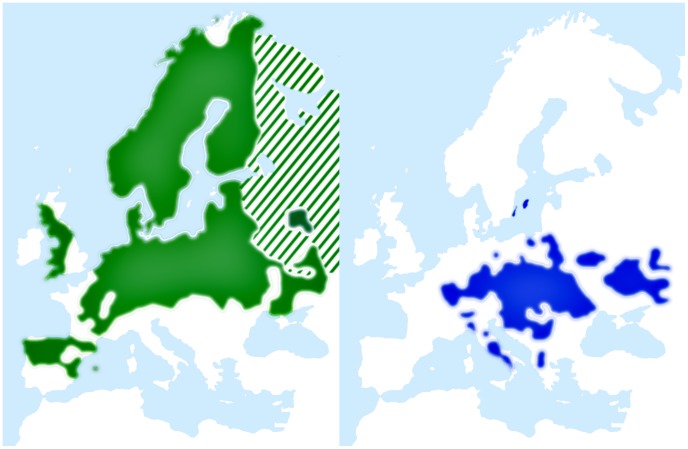
Breeding range distributions of pied flycatcher (green) and collared flycatcher (blue). Maps adapted and redrawn from EBCC (European Bird Consensus Council) Atlas of European Breeding Birds (http://s1.sovon.nl/ebcc/eoa/). Stripes indicate uncertainty of species existence in the area.

We have recently sequenced and *de novo* assembled the 1.1 Gb genome of the collared flycatcher *Ficedula albicollis* [57]. Together with its sister species, the pied flycatcher (*F. hypoleuca*), it forms an important model system in evolutionary ecology and biology (e.g. [58]–[61]), including studies of hybridization and speciation [62], [63], and genetics [64]–[70]. The two flycatchers are small, migratory birds that belong to the order Passeriformes. The pied flycatcher breeding range covers a large part of the western Palearctic (Figure 1) and overlaps with collared flycatcher in two areas (central Europe and Baltic Sea islands). In these regions the species coexist and hybridize occasionally [71]. However, the fitness of hybrid offspring is severely reduced [72], with females apparently being sterile [73]–[76]. This is in accordance with Haldane's rule, as birds have female heterogamety. Previous genetic studies in flycatchers have indicated no or very low levels of gene flow between allopatric populations of pied flycatchers and collared flycatchers, and moderate gene flow in the area of recent sympatry on the Baltic islands [28], [77], [78].

Here we capitalize on data from a whole-genome re-sequencing effort in flycatchers [57]. These data, comprising >10 million single nucleotide polymorphisms (SNPs), allow us to carefully choose genomic regions spread across the flycatcher genome and analyze them in an ABC framework (augmented by PSMC modeling) to infer demography and gene flow during different stages of species divergence in this ecological model system.

## Results

### Patterns of Genetic Variation

After stringent filtering of whole-genome re-sequencing data from 10 collared flycatchers and 10 pied flycatchers, each individual sequenced at an average of 5× coverage, we investigated sequence variation in 267 independent, noncoding loci covering a total of 534 kb (≈0.05% of the genome). At these loci, genotypes could be called at 429,753 sites in at least seven individuals in each species. Sequence diversity was higher in collared flycatcher (mean π = 0.0033±0.0034) than in pied flycatcher (mean π = 0.0020±0.0025). The data contained a substantial fraction of shared polymorphisms (i.e., sites segregating in both species; 0.22±0.02) and some fixed differences (0.03±0.00); note that ‘fixed’ in this context means monomorphic for different alleles in these particular samples of the two species. We observed many more SNPs that were unique to collared flycatcher (0.54±0.02; fraction of all SNPs) than to pied flycatcher (0.21±0.02). The differentiation between species was moderate (mean F_st_ = 0.21±0.01). Mean values for Tajima's D statistics were positive for both species (0.17±0.28 for collared flycatcher; 0.40±0.43 for pied flycatcher). All summary statistics are in good agreement with genomic background variation recently reported for whole-genome data [57].

### Model Choice Procedures

**Figure 2:**
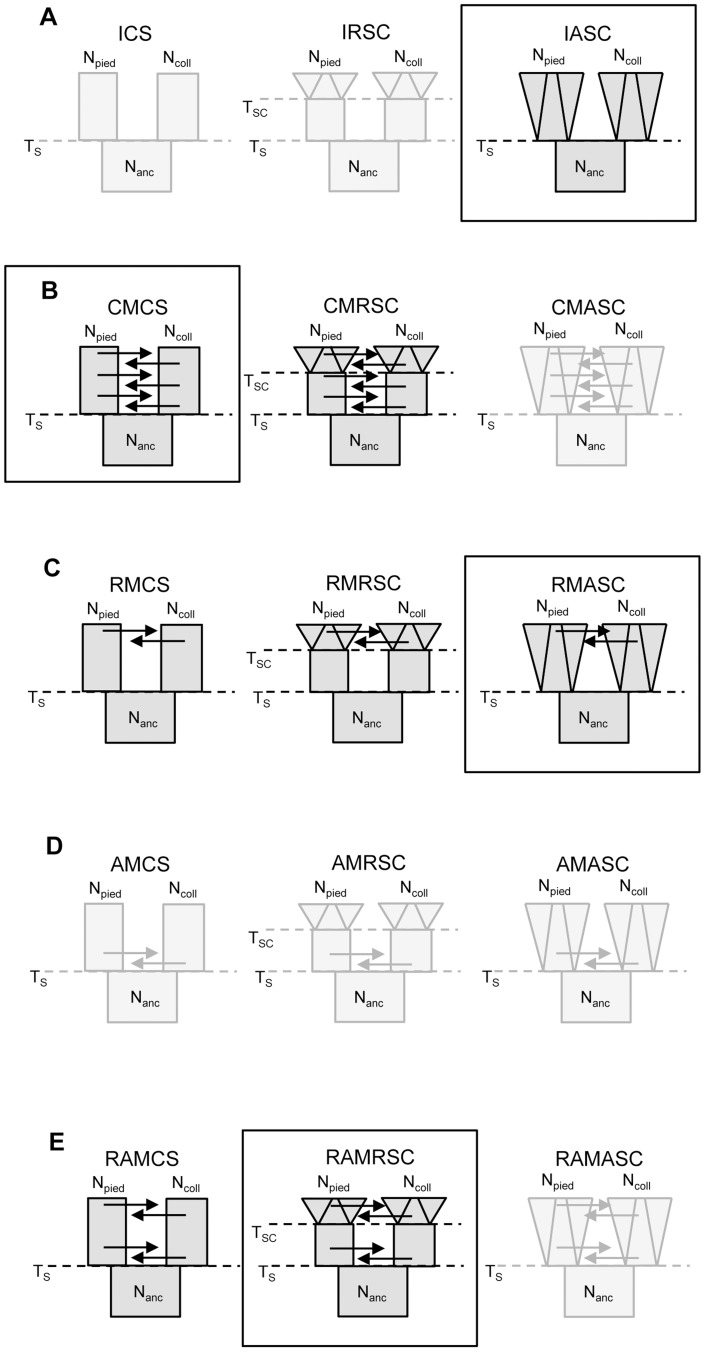
Scenarios and models investigated in the study. A – isolation scenario (ICS – ‘isolation constant size’, IRSC – ‘isolation recent size change’, IASC – ‘isolation ancient size change’); B – constant migration scenario (CMCS – ‘constant migration constant size’, CMRSC – ‘constant migration recent size change’, CMASC – ‘constant migration ancient size change’); C – recent migration scenario (RMCS – ‘recent migration constant size’, RMRSC – ‘recent migration recent size change’, RMASC – ‘recent migration ancient size change’); D – ancient migration scenario (AMCS – ‘ancient migration constant size’, AMRSC – ‘ancient migration recent size change’, AMASC – ‘ancient migration ancient size change’); E – recent and ancient migration scenario (RAMCS – ‘recent and ancient migration constant size’, RAMRSC – ‘recent and ancient migration recent size change’, RAMASC – ‘recent and ancient migration ancient size change’). The models shown in light grey are the models for which the likelihood of observed data did not fall within the distribution of simulated data. The frames indicate the best model of each scenario. N_pied_ and N_coll_ – effective population size of pied flycatcher and collared flycatcher, respectively; N_anc_ – ancestral population size.

We examined 15 demographic models of flycatcher divergence (five scenarios with three models each; Figure 2), and identified eight models for which the likelihood of observed data (calculated under Generalized Linear Model) fell well within the distribution of retained simulated data (Table S1). These included models from four demographic scenarios: three models from a scenario with recent gene flow (‘recent migration constant size’, RMCS; ‘recent migration recent size change’, RMRSC; recent migration ancient size change, RMASC), two models from a scenario with constant migration (‘constant migration constant size’, CMCS; ‘constant migration and recent population size changes’, CMRSC), two models from a scenario with ancient and recent migration with a period of isolation between the two phases of gene flow, either with constant population size (RAMCS), or recent population size changes (RAMRSC) and one model without migration between species (‘isolation ancient size change’, IASC). All models with ancient gene flow yielded very low P-values (most of the simulated datasets having much higher likelihood than the likelihood of the observed data), indicating that they did not fit the observed data.

**Figure 3:**
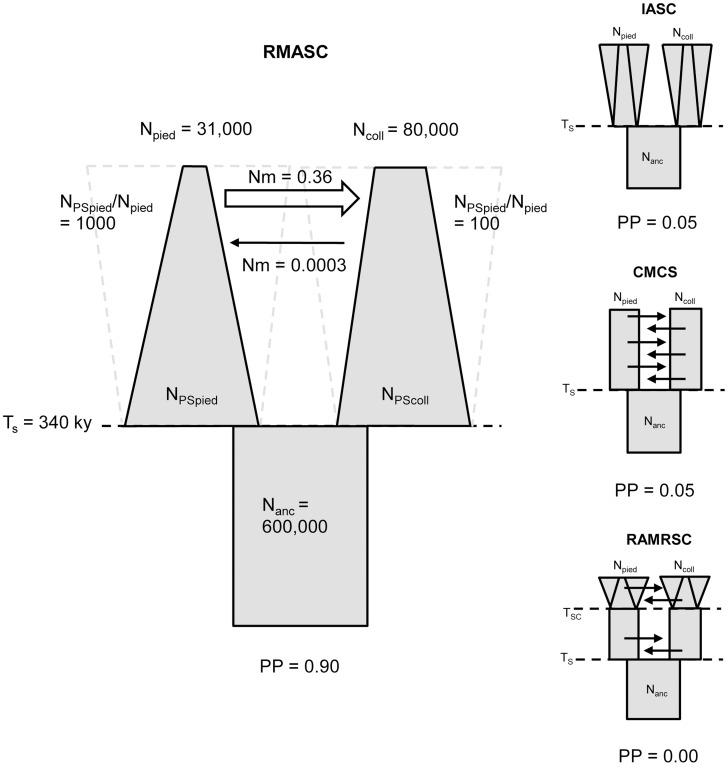
The four best models and their posterior probabilities (PP). Recent migration and ancient size change model (RMASC) was chosen for the full estimation procedure. N_pied_ and N_coll_ – effective population size of pied flycatcher and collared flycatcher, respectively; N_PSpied_ and N_PScoll_ – effective post-split population size of pied flycatcher and collared flycatcher; N_anc_ – ancestral population size. T_S_ – time of split; Nm – number of migrants per generation.

Model choice conducted within each of the four plausible scenarios suggested four models that fit the observed data best: IASC, CMCS, RMASC and RAMRSC (Figure 3). Of these models RMASC had the highest posterior probability (PP = 0.90); the posterior probabilities for IASC, CMCS and RAMRSC were very low (0.05, 0.05 and 0.00, respectively). The RMASC model was clearly the best model also when we compared the eight models that fit the data well in a single model selection procedure (PP = 0.77) as well as when we used an alternative nesting procedure (migration nested within population size dynamics; PP = 0.98). The RMASC model was also the best model when ‘not-optimized’ prior ranges were used suggesting that the choice of prior ranges had little influence on best model selection (Table S2). The power to correctly predict the models was 0.57, 0.83, 0.67, and 0.74, which is much higher than the expected 25% and indicates that we were able to clearly discriminate the models. RMASC, i.e. the model with recent migration and ancient size change, fitted the data significantly better than all other tested models, and was therefore chosen for parameter estimation (Figure 3).

### Validation of the Best Model and Parameter Estimation

**Figure 4:**
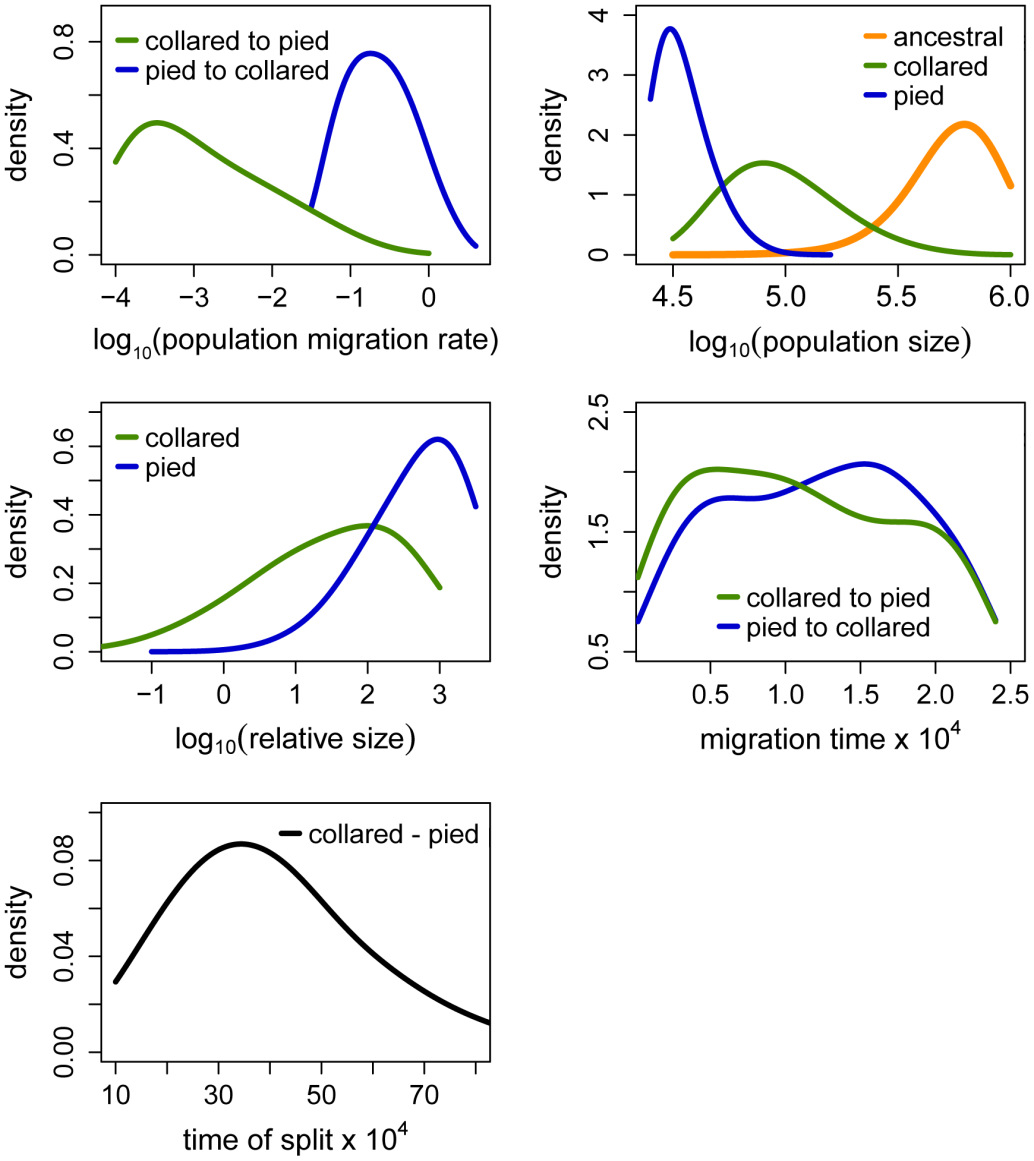
Posterior probabilities of key parameters of the recent migration and ancient size change (RMASC) model. The term ‘relative size’ refers to the ratio of post-split effective population size and current effective population size (e.g. N_PScoll_/N_coll_). Population migration rate equals to 4N_0_m_ij_, where N_0_ = 10,000.

**Figure 5:**
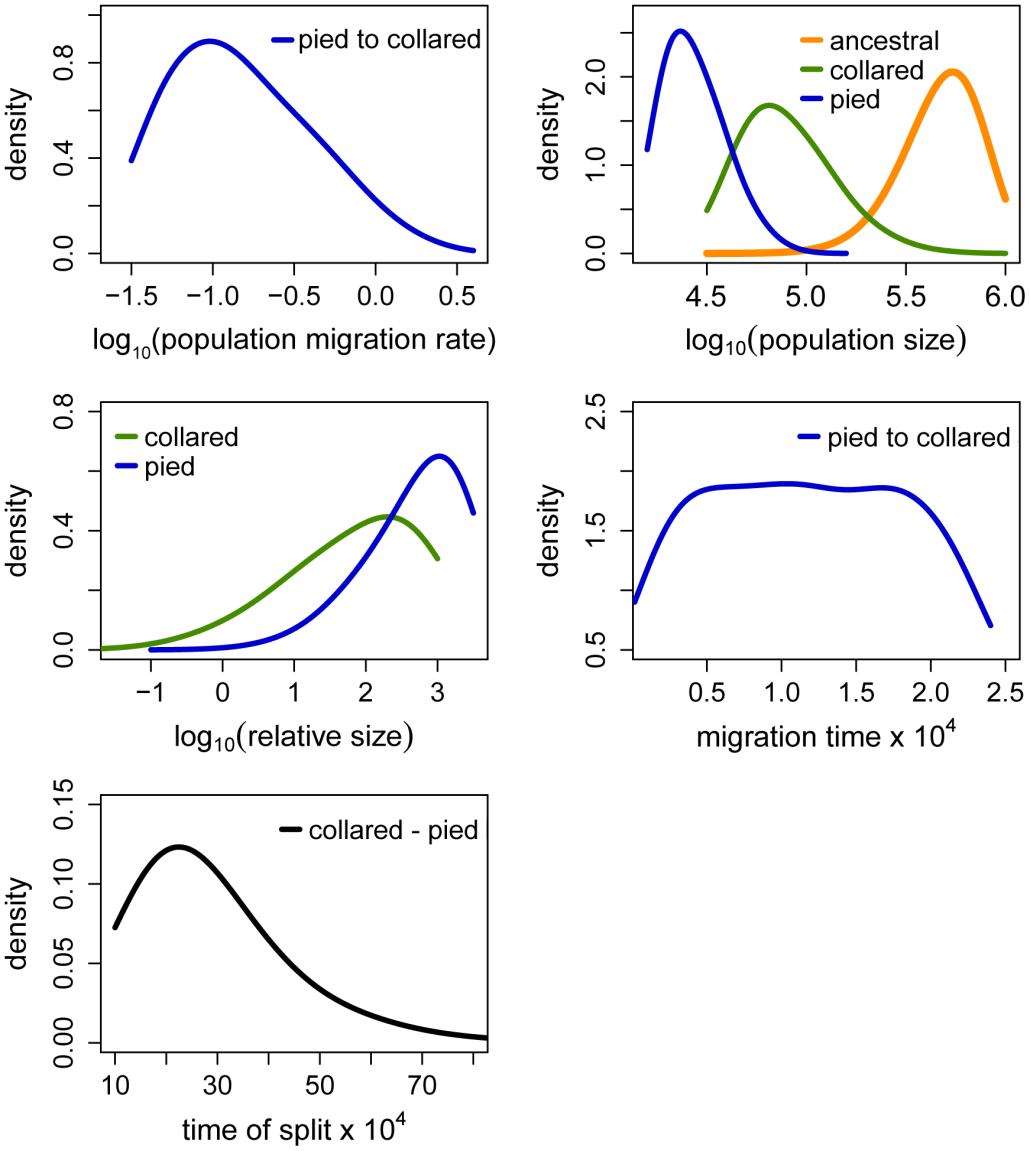
Posterior probabilities of key parameters of the recent unidirectional migration and ancient size change (RUMASC) model. The term ‘relative size’ refers to the ratio of post-split effective population size and current effective population size (e.g. N_PScoll_/N_coll_). Population migration rate equals to 4N_0_m_ij_, where N_0_ = 10,000. Due to smoothing step of the parameter distributions the densities at the prior limits are underestimated.

The Partial Least Squares (PLS) components of the observed summary statistics fell well within the density distribution of the PLS components of the retained simulations, demonstrating that simulations were appropriately exploring the summary statistic space (Figure S1). To verify the coverage properties of the marginal posterior distributions estimated with the chosen estimation approach, we generated 1,000 pseudo-observed data sets and tested the distributions of posterior quantiles for each parameter of the best model (based on 15,000 retained simulations and seven PLS components). Most of the parameters had a uniform distribution (Table 1; Figure S2) and coefficients of variation indicated that we had enough power to estimate most of the parameters (R^2^>10%; [79]; Table 1). Nevertheless, to reduce complexity we also considered a model assuming no migration from collared flycatcher to pied flycatcher. This was motivated by very low amounts of gene flow in this direction estimated in the RMASC model (mode = 8.33×10^−9^). We also updated priors for effective population size of pied flycatcher based on posterior distributions. The model with unidirectional migration (model RUMASC) was run for 2×10^6^ simulations, and submitted to careful examination and validation based on 5,100 retained simulations and seven PLS components. The RUMASC model had higher posterior probability (PP = 0.62) than the RMASC model (PP = 0.38), and the distribution of posterior quantiles exhibited limited bias in the posterior distributions (Table S3; Figure S3). However, the power to correctly predict models RMASC and RUMASC was rather small (0.58 for RMASC and 0.63 for RUMASC). This is expected since both of them produced very similar posterior probability distributions. We therefore present parameter estimates for both models (Figure 4, Figure 5, Table 1, Table S3 and Table 2).

**Table 1:** Prior and posterior distributions of recent migration and ancient size change (RMASC) model.

	Prior^a^		Estimation validation			Posterior characteristics						
							HPDI 50		HPDI 90		HPDI 95	
Parameter	minimum	maximum	P value^b^	R^2^ c	RMSE^d^	Mode	Lower	Upper	Lower	Upper	Lower	Upper
log_10_(N_coll_)	4.5	6	0.116	0.56	0.32	4.90	4.74	5.09	4.56	5.39	4.52	5.48
log_10_(N_pied_)	4.4	5.2	0.86	0.41	0.19	4.49	4.43	4.57	4.40	4.73	4.40	4.80
log_10_(N_anc_)	4.5	6	0.139	0.45	0.33	5.80	5.67	5.91	5.44	6.00	5.33	6.00
log_10_(N_PScoll_/N_coll_)	−3	3	0.096	0.32	1.46	2.01	1.12	2.56	−0.2	2.98	−0.69	2.98
log_10_(N_PSpied_/N_pied_)	−1	3.5	0.047	0.5	0.93	2.98	2.5	3.35	1.53	3.49	1.17	3.49
log_10_(M_pied→coll_)^e^	−1.5	0.6	0.072	0.47	0.62	−0.74	−1.05	−0.37	−1.38	0.11	−1.46	0.21
log_10_(M_coll→pied_)^f^	−4	0	0.007	0.25	0.94	−3.48	−3.89	−2.8	−3.99	−1.49	−3.99	−1.13
Tm_pied→coll_	150	25000	**0.002**	0.11	0.15	15255	8780	19028	2137	22088	1214	22862
Tm_coll→pied_	150	25000	0.05	0.01	0.19	5431	2496	12768	1059	21362	460	22305
T_s_	10000	1000000	0.015	0.23	5.81	344222	228895	476056	106785	686688	106785	777128
μ×10^−9^	1	5	**0.002**	0.43	0.89	1.38	1.1	1.71	1.01	2.46	1.01	2.76
*r*×10^−8^	0.1	10	0.01	0.08	2.62	8.21	6.1	9.38	2.95	9.98	2.1	9.98

a all priors are uniformly distributed.

b P value computed with Kolmogorov-Smirnoff test; bold values indicate significant deviations from uniformity after Bonferroni correction.

c coefficient of determination.

d average root mean square error.

e M_pied→coll_ equals 4N_0_m_pied→coll_; N_0_ = 10,000.

f M_coll→pied_ equals 4N_0_m_coll→pied;_ N_0_ = 10,000.

**Table 2:** Key parameter values of RMASC and RUMASC models.

Parameter	Mode		Lower HPDI 90		Upper HPDI 90	
N_coll_	79,341	64,424	36,650	33,025	246,315	182,188
N_pied_	30,792	23,218	25,235	16,126	54,282	48,233
N_anc_	625,864	544,728	272,929	247,298	991,357	1,004,570
N_PScoll_/N_coll_	101	203	1	2	966	966
N_PSpied_/N_pied_	955	1,060	34	34	3,081	3,081
m_pied→coll_	4.55×10^−06^	2.42×10^−06^	1.05×10^−06^	8.20×10^−07^	3.21×10^−05^	1.77×10^−05^
m_coll→pied_	8.33×10^−09^		2.56×10^−09^		8.17×10^−07^	
Tm_coll→pied_	15,255	10,343	2,137	1,657	22,088	21,734
Tm_pied→coll_	5,431		1,059		21,362	
T_S_	344,222	226,634	106,785	102,262	686,688	524,476

Distributions of divergence time (T_S_) estimates fell within the range of a few hundred thousand years indicating recent origin of the flycatcher species (mode T_S_≈340,000 years in RMASC and 230,000 years in RUMASC). The estimated population size of the common ancestor (mode N_anc_≈600,000 and 550,000, respectively) was much larger than current N_e_ of both collared flycatcher (mode N_coll_≈80,000 and 65,000, respectively) and pied flycatcher (mode N_pied_≈31,000 and 23,000, respectively). Both species thus showed a strong signal of population decline since their initial divergence, with the decrease being more severe in the pied flycatcher than in the collared flycatcher. Posterior probability curves of the relative size of post-split and current population size (N_PScoll_/N_coll_ and N_PSpied_/N_pied_) encompassed only values larger than one, but the strength of the decline is difficult to estimate due to wide 90% highest posterior density intervals (HPDI). The rate of gene flow from collared flycatcher to pied flycatcher was very low (RMASC, mode = 8.33×10^−9^). In the opposite direction, m_pied→coll_, gene flow was estimated 4.55×10^−6^ in RMASC and 2.42×10^−6^ in RUMASC. This corresponds to 0.36 and 0.16 migrants per generation, or one migrant about every three and six generations, respectively. Although the exact timing of gene flow between populations was not possible to estimate (very wide and flat posterior probability distributions of Tm_coll→pied_ and Tm_pied→coll_), a model with recent (after Last Glacial Maximum, LGM) gene flow was favored.

### PSMC-Based Estimation of Population Size Change in Collared Flycatcher

**Figure 6:**
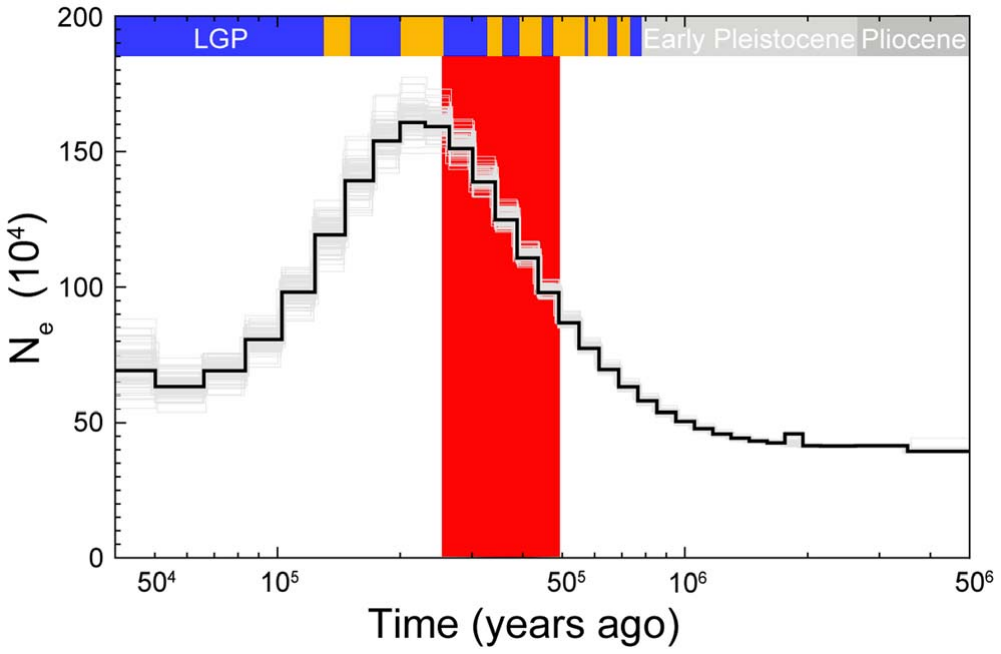
PSMC estimate of the effective population size change over time for collared flycatcher. The black curve is the PSMC estimate for the original data and the grey curves indicate PSMC estimates for 100 bootstrapped sequences. Glacial and interglacial periods of the Late and Middle Pleistocene are indicated by blue and yellow bars, respectively. The interglacial periods corresponds to Marine Isotope Stages: 5e, 7, 9, 11, 13, 15, and 17. The large red-shaded area corresponds to 50% HPDI of the time of divergence (RMASC model). LGP – last glacial period.

To investigate changes in N_e_ over time in more detail we performed pairwise sequentially Markovian coalescent modeling (PSMC) analysis using the diploid sequence of a collared flycatcher male sequenced at 85× coverage. The analysis showed good resolution between 50 ky and 2 my, and rather small variance associated with most of the N_e_ estimates (Figure 6). The effective size of the population substantially increased from approximately 500,000 individuals 1 my ago (i.e., before the pied flycatcher-collared flycatcher split) to a maximum of 1.6 million individuals 200 ky ago. From approximately 200 ky ago effective size started to decrease and reached about 500,000 individuals several tens of thousands years ago. The ABC estimate of the effective size of the ancestral population (≈700,000) was thus very similar to the PSMC estimate of N_e_ before species divergence. Due to a limited number of recent coalescent events that can be inferred from a single genome sequence, the estimation of more recent changes in N_e_ is not possible [15].

## Discussion

### Demographic History of Species Divergence in Pied Flycatcher and Collared Flycatcher

We analyzed sequence variation in several hundred intergenic loci (totaling ≈0.5 Mb) to infer demographic parameters of the divergence history of pied flycatcher and collared flycatchers. Stringent filtering of whole-genome re-sequencing data and careful evaluation of ABC analyses enabled us to infer the demographic scenario of species differentiation with high confidence. The divergence time estimate was consistent with a recent, middle Pleistocene split of the common ancestor of the two species. Since their initial divergence N_e_ of both species declined and unidirectional gene flow from pied flycatcher into collared flycatcher took place at a recent time scale (most likely after the LGM). Some, but not all, demographic parameters were in good agreement with previous estimates [28], [77]. However, in addition to previous studies that were based on limited data and simple demographic models (constant migration, no population size change over time; [28], [77]), our genome-wide approach enabled us to study divergence in much greater detail. We explicitly modeled contrasting patterns of gene flow and population size changes over time and our results consequently reveal new and important demographic aspects of the divergence history of pied flycatcher and collared flycatcher, which contribute to understanding of the genomic landscape of species divergence in this system. Phrased differently, the work can be seen as relevant in the context of genome divergence as well as of species divergence.

The estimated effective size of the ancestral population (≈600,000) was larger than the current N_e_ of both species, and much larger than the ancestral N_e_ estimate of 130,000 reported by Backström at al. [28] (see further below). In agreement with observed patterns of intraspecific diversity, current N_e_ of collared flycatcher (65,000–80,000) was higher than that of pied flycatcher (23,000–31,000), similar to values estimated for other European populations of these species [28], [77]. However, N_e_ estimates of both species are in sharp contrast to estimated census sizes in Europe. These are two to three orders of magnitude larger, with 4.2–7.2 million for collared flycatcher and 36–60 million for pied flycatcher (http://www.birdlife.org). The remarkable discrepancies between census and effective population sizes indicate successful postglacial expansions from apparently significantly bottlenecked refugial populations in both species. Moreover, the much higher census size of pied flycatcher compared to collared flycatcher coupled with the opposite relationship for N_e_ suggests a more rapid, and as testified by current breeding ranges, more extensive post-glacial re-colonization of northern habitats by pied flycatcher. This is in line with the estimated relative sizes of post-split and current N_e_ (N_PScoll_/N_coll_≈100 and N_PSpied_/N_pied_≈1000) of both species, which indicate a much more severe decline for pied flycatcher. As a general caveat to these issues, we note that changes in population structure over time may affect coalescent rate estimates and, as a consequence, influence N_e_ estimates [21].

### Complementing ABC-Inference with PSMC Analysis

Although the ABC-based estimation of the magnitudes of population decline has to be treated with some caution, our analyses confidently evidence significant post-divergence population size decreases in both species. The rank order for N_e_ (N_PScoll_ and N_PSpied_>N_anc_>N_coll_ and N_pied_) indicates that the ancestral population differentiated into two descendent populations without any sign of bottleneck associated with initial divergence. Both post-split populations appear to first have increased in size before subsequent population decline during glacial periods. While this interpretation would remain speculative based on the ABC analyses alone, it is supported by the PSMC results. The time of population size increase in the PSMC curve for collared flycatcher largely overlaps with divergence times estimated by ABC, indicating an increase in collared flycatcher N_e_ after initial differentiation from the ancestral population (Figure 6). The mode for T_S_ in RMASC model (340,000) and its 50% HPDI (230,000–480,000) include almost exclusively the epoch before N_e_ decline indicated by the PSMC curve. Assuming that the maximum N_e_ from PSMC analysis (1.6 million) approximates N_PScoll_, the ratio of N_PScoll_/N_coll_ indicates a 20-fold decline in population size in the last 200 ky (N_PScoll_/N_coll_ = 20.17). This value falls well within the 50% HPDI estimated by ABC analysis, lending additional support for the RMASC model and ABC-based estimates. On the other hand, the mode of T_S_ from RUMASC model (230,000) coincides with the peak of the PSMC curve. However, it is important to note that the divergence time estimate in this model can be biased (as indicated by distribution of posterior quantiles, Table S3) and has to be treated with caution. Nevertheless, regardless of the divergence time estimates and consistent with ABC analysis, PSMC estimation of N_e_ clearly indicates a rapid population decline during the first half of the last glacial period (100,000 - 50,000 years ago). An alternative scenario consistent with PSMC estimates would imply a population split of the post-split collared flycatcher population into two or more subpopulations followed by their admixture after a period of isolation. In this case PSMC as well as other coalescent-based methods may overestimate N_e_ during the period of population split (Li and Durbin 2011).

### Recent Unidirectional Gene Flow from the Resident into the Colonizing Species

In addition to the evaluation of population size changes during species divergence, the ABC approach enabled us to model various scenarios of gene flow over time. A model with gene flow occurring exclusively after the LGM was favored, with an estimate equivalent to one individual per three to six generations introgressing from pied flycatcher to collared flycatcher. In accordance with previous studies [28], [77], the rate of gene flow in the inverse direction, from collared flycatcher to pied flycatcher was estimated as essentially absent. The estimated pattern of gene flow is in line with the expectations for invading and resident populations: unidirectional gene flow from the resident, stable population into the expanding, invading population [80], [81]. Thus, it is most likely that pied flycatcher colonized northern Europe more rapidly than collared flycatcher and collared flycatcher arrived some time later as an invading species. The scenario is supported by the patterns of estimated N_e_ (discussed above) and also by recent observations form the Baltic Sea islands where collared flycatchers colonized habitats previously inhabited only by pied flycatchers [82].

### Rapid Evolution of Reproductive Isolation despite Short Divergence Time

Interestingly, the estimates of the divergence time between species (modes of 340,000 and 230,000 years ago in RMASC and RUMASC, respectively) indicate much more recent divergence than estimated from mitochondrial DNA (mtDNA; 1–2 my based on ≈3% mtDNA divergence; [64]). Since pied flycatcher and collared flycatcher have already reached an advanced stage of reproductive isolation (female hybrids are sterile, male hybrids have significantly reduced fertility; [63], [72]) this may be seen as surprising given that birds are thought to develop reproductive barriers rather late in the speciation process [83]. However, mtDNA-based estimates of divergence time may be biased for at least two reasons. First, gene divergence often predates species divergence [84]. Second, due to the stochastic nature of the coalescent process and huge variance associated with single-locus estimates of TMRCA, estimates of divergence time based on mtDNA alone might be unreliable [85]. Indeed, a model-based approach applied to mtDNA data would give huge credible intervals. Divergence time estimates based on 24 autosomal loci clearly reduced variation related to the coalescent processes and pointed towards more recent divergence (approximately 0.5 my; [28]). However, the distribution of IMa-based divergence time was still wide with 90% HPDI exceeding 1 my. With the resolution now given by the genome-wide approach, we could further narrow the interval to less than 700,000 years.

Although the flycatcher system may be exceptional when it comes to the rate of formation of reproductive incompatibility, we note that the hypothesis of speciation potentially being a relatively slow process in birds, with extended times for intrinsic postzygotic reproductive barriers to evolve, is mainly based on data from mtDNA studies [83], [86]. If other genome-wide studies of avian speciation models will also come to suggest more recent divergence than estimated by data from mtDNA, and some preliminary data actually point in this direction (e.g. [87], [88]), this hypothesis may have to be revised. We also note that our estimates of divergence time derive from the ability to include population size changes in ABC models, which has not been possible in previous work. Ignoring the detected decline would lead to an upwards biased divergence time estimate.

### The Advantage (and Limitation) of Using Genome-Wide Data and Genome Annotation

Differences between our results and the results presented by Backström et al [28] and Hogner et al. [77] seem most likely attributable to the fact that previous work was based on a relatively limited number of intronic loci. The increased amount of data in the present study may thus have contributed to substantially improving the accuracy of demographic parameter estimates [89]. Moreover, the general pattern of variation observed in our genome-wide data differed from the previous intronic datasets. Consistent with Ellegren at al. [57], nucleotide diversity was smaller than estimates based on limited intronic data (mean π = 0.0020 and 0.0033 for pied flycatcher and collared flycatcher in our genome-wide data and 0.0041 and 0.0044 for populations studied in [28]). This explains our lower estimates of N_e_ for both species. Also, while mean Tajima's D was positive for collared flycatcher in genome-wide data (0.17), it was estimated negative in previous studies (−0.32).

The likelihood of different scenarios of gene flow during speciation is currently a much debated topic in evolutionary biology (e.g. [3], [4], [90]). Besides the mere verification whether speciation can occur in the face of gene flow, a challenging task is to distingiush between scenarios with gene flow already during initial differentiation (sympatric or parapatric speciation), constant migration during divergence (or multiple admixture events), and gene flow occurring only after a long period of allopatric divergence when populations come into secondary contact. By explicit modeling of different patterns of gene flow over time, we were able to infer a demographic history consistent with allopatric speciation followed by secondary and recent contact as the most likely scenario of flycatcher differentation (models with ancient gene flow had very low posterior probabilities). The results are important in the context of the overall genomic landscape of species divergence in this system. We have recently shown the genomic landscape is highly heterogeneous with one or a few regions per chromosome showing highly elevated differentiation (divergence peaks, potentially representing “genomic islands of speciation”; [57]). These regions, which are low in shared polymorphisms between species and high in private polymorphisms (relative to other regions of the genome), are candidates to have evolved under the strong influence of selection. With the relatively recent divergence suggested by our analyses (i.e. mode of 340,000 and 230,000 my, respectively), elevated divergence in these islands must have been built up rapidly. The co-localization of divergence peaks and centromeres as well as telomeres fed the hypothesis that meiotic drive may have been involved in generating high divergence [39] and potentially segregation distortion is a process potent enough to rapidly generate genetic incompatibilities. Also, reduced recombination in centromeres may contribute to high divergence, however, recombination at telomeres seems elevated on avian chromosomes [91].

It is possible that gene flow upon secondary contact reinforces a genomic landscape of heterogeneous sequence divergence. Specifically, introgression may lower background levels of divergence, or at least act as to their maintenance, while selection, if it occurs, continues to build up divergence in genomic islands in which gene flow is hindered. Importantly, with the levels and continuance of gene flow observed, gene flow alone cannot have been sufficient to have had a predominant role in the evolution of the differentiation landscape. The general implication of this is that the differentiation islands have to be explained by different mechanisms than the breaking down of differentiation by gene flow in the genomic background. As indicated above, a candidate mechanism is obviously divergent selection, however, locally enhanced lineage sorting due to a heterogeneous recombination landscape cannot be excluded and will require very fine-scale estimates of recombination rates to be addressed.

While our study provides unusually detailed insight into the demographic history and the processes affecting genetic differentiation at a genome-wide scale in a speciation model system, it should be stressed that most posterior estimates still reflect considerable uncertainty. Even with the access to a draft genome assembly, data from whole-genome re-sequencing of population samples and complex and computer intensive methods, there are thus limitations as how far genetic data can perfectly reconstruct demographic history. Some accuracy may have been gained by analyzing additional loci, however, theoretical work has recently shown that the width of credible intervals in ABC analyses rapidly decreases when hundreds of loci are analyzed [89]. Moreover, adding more loci would have required relaxing the criterion of only including loci located at least 500 kb apart, which would have made linkage disequilibrium (LD) a possible issue. For future studies, summary statistics taking into account the structure of LD may represent the most promising avenue in order to further distinguish between scenarios and by improving parameter estimates. However, this can only be obtained with sufficient confidence from higher-coverage re-sequencing data than used herein.

We investigated the demographic history of two closely related bird species using whole-genome re-sequencing data and a full ABC approach supported by additional coalescent-based analysis. By applying stringent filtering, careful ABC evaluation and hierarchical model choice we were able to investigate different demographic scenarios including different patterns of population size change and gene flow over time. The best-supported scenario of flycatcher divergence indicated that the ancestral species survived one of the glacial periods of middle Pleistocene, split into two large populations that both appear to have increased in size during the warm interglacial period before they experienced severe bottlenecks. The species probably came into secondary contact after LGM, which resulted in mostly unidirectional gene flow from pied flycatcher to collared flycatcher. Our study constitutes one of the first examples of detailed modeling of the complex divergence history in an emerging model system for speciation genomics. Indeed, *Ficedula* flycatchers may be a type example of speciation during Pleistocene, where alternating cycles of glacial and inter-glacial periods have shaped genomic differentiation.

## Materials and Methods

### Samples and Loci

We randomly sampled independent loci distributed across the genome, each comprising 2,000 bp of assembled sequence. Each locus was required to be situated at least 500 kb apart from other sampled loci. This physical distance is well above the lengths of linkage disequilibrium blocks seen in collared flycatcher [68]. First, we randomly sampled 2,000 loci (the maximum amount in an approximately 1 Gb genome theoretically possible when not allowing loci to be closer than 500 kb apart) and were able to collect 1,086 loci fulfilling the density criterion, a reduction following from randomness of sampling and chromosome structure. For further analysis we only kept loci that were found in autosomal, noncoding regions of the genome, and we excluded sequences that exhibited elevated levels of divergence (“divergence islands” identified in [57]). Sequence data were subsequently extracted for 10 pied flycatcher and 10 collared flycatchers (generated as described in [57]) and filtered based on sequence coverage. For every sampled locus we analyzed only those sites that passed the threshold of being covered by at least 3 reads per site in at least seven individuals per species; all sites that did not pass the filter's threshold were masked as missing data. This strategy should have enabled us to filter out most of the sequencing errors. As a next filtering step, we used only loci that consisted of no more than 30% of missing data. This step reduced computational time by not simulating too many sites that would not be used in further inference. To avoid the risk of mistakenly calling a heterozygous site as homozygote we haploidized sampled loci by randomly sampling one allele per site. Our final dataset contained 267 loci, i.e. all loci fulfilling all criteria, at which 80% of the sites (429,753 bp out of a total of 534 kb) were covered by called genotypes for each individual.

### Approximate Bayesian Computation

We analyzed the data under an Approximate Bayesian Computation framework [32]. ABC methods come in different flavors but all standard approaches share the same general scheme and strategy: 1) The observed data is characterized by a set of summary statistics known to be informative about parameters of interest, 2) millions of datasets are generated under a demographic model, each with different parameter values randomly drawn from given prior distributions, 3) if more than one model is considered, the best fitting model is selected, and 4) datasets for which summary statistics are closest to those obtained from real data are used for estimation of the best model parameters [39], [40]. Although intuitively straightforward, ABC is not a ‘plug and play’ analysis and often requires careful investigation of each step of the protocol and several quality control checkpoints.

We used the *ABCtoolbox* software designed to perform ABC analysis and facilitate and integrate simulation, summary statistics calculation, and parameter estimation steps into a single pipeline ([92]; kindly updated for us by D. Wegmann). Simulations were performed using *msABC* [93], a modified version of *ms* [94]. Since we stringently filtered sequence data, we paid special attention to treat the simulated data in the same way. Thus, in every *msABC* iteration we simulated 267 loci (for 10 pied flycatchers and 10 collared flycatchers), masked all sites that did not pass the coverage threshold in the original data (see above in Samples and Loci) and, before calculating summary statistics, haplodized the data.

### Prior Model Parameter Distributions

As required in *msABC* all parameters were scaled by a factor N_0_ which we set to 10,000. Thus, effective population size (N_e_) was simulated as N/N_0_; time parameters equalled T/4N_0_ and migration parameters (M) were scaled as 4N_0_m_ij_, where m_ij_ is the fraction of population i which is made up of migrants from population j each generation. We based most of the parameter prior range distributions on the results from previous studies (IM estimates based on 24 nuclear loci; [28]) and kept them wide enough to ensure that a variety of plausible parameter values could be captured. We used a standard ABC approach and sampled parameter values from uniform distributions and for most cases set to a log_10_ scale (N_e_, relative population sizes, migration rates. Recombination rate (*r*) prior was set based on a high-density recombination map recently developed for the collared flycatcher (unpublished data); for each simulated locus we obtained the local estimate of the recombination rate (mean 5.3×10^−8^). Based on the distribution of recombination rates we set the recombination rate for each locus to be drawn from a Gamma distribution G (α,α/*r*), with the shape parameter α drawn from U [1], [12]. The mean mutation rate (μ) prior was chosen based on our previous estimates [57], [95]. The mutation rate for each locus was then drawn from a Gamma distribution G(α,α/μ), with the shape parameter α drawn from U [3], [12].

### Demographic Models

To start with, we first ran five classes of exploratory simulations including different demographic scenarios: isolation, constant migration over time, recent (after LGM) migration, ancient migration, and ancient as well as recent migration with a period of isolation between two phases of gene flow (Figure 2). For each scenario we investigated three models in which we either assumed 1) constant N_e_ of descendant populations, 2) exponential change in N_e_ of descendant populations after the LGM, or 3) exponential change in N_e_ of descendant populations since their initial divergence. Population size changes were modeled by assuming that the population size at the start of size change was a fraction x of the current N_e_ (priors were set to capture both population growth and decline). In all models with recent migration we assumed bidirectional migration and the ranges of priors were set to cover very small to moderate levels of gene flow. Since the ability to detect strong signatures of asymmetric gene flow between ancient populations is very low we assumed symmetrical migration in all models with ancestral migration events. The number of exploratory simulation varied from 100,000 to 200,000, enough to judge if the model is able to explain the observed data. For every model we checked the fraction of retained simulations (2.5%) with a smaller or equal likelihood than the likelihood of the observed data (P-value reported by *ABCtoolbox*). The likelihoods were estimated for truncated models under General Linear Model post-sampling adjustment (ABC-GLM, [38]). We also inspected the posterior probability curves to check if the model fitting could be improved by changing the ranges of priors. In several cases we updated ranges and ran particular scenarios one more time. All models for which the likelihood of observed data fell within the distribution of simulated data were run for 2 million simulations.

### Choice of Summary Statistics

ABC inference was based on a set of summary statistics calculated for each species separately and for both species combined. We calculated mean and variance across all 267 investigated loci using *msABC* for the following summary statistics: nucleotide diversity (*π*), Tajima's D (D) and F_st_. In addition, using in-house perl scripts we calculated the proportions of shared, fixed, private (for pied flycatcher and collared flycatcher, respectively) polymorphisms. Following Wegmann at al. [18], we defined a set of orthogonal linear-combinations of summary statistics that best explained the variance in the model parameter space by transforming the full set of summary statistic via Partial Least Squares [96]. All transformation were done in the *R* package *PLS* [97] and the appropriate number of PLS components were defined based on root mean squared error plots (RMSEP plots). PLS transformed statistics were used to calculate the Euclidean distance between observed and simulated datasets and up to 3% of simulations with the smallest distance were retained for parameter estimations via the regression adjustment ABC-GLM [38] implemented in *ABCtoolbox*.

### Model Choice

The model choice procedure was conducted in the *ABCtoolbox*. We used distances calculated based on PLS components to choose the simulations that were closest to the observed data but untransformed summary statistics dataset (excluding statistics that were highly correlated: mean and variance of number of fixed differences correlated with mean F_ST_ and variance of number of private polymorphisms (respectively) in IASC model; mean and variance of nucleotide diversity for both species correlated with pied flycatcher nucleotide diversity estimates in CMRSC model) to perform model selection via Bayes factors (ratios of marginal densities). Following Fagundes at al. [98] we applied a hierarchical model choice procedure. First, we evaluated posterior probabilities of different models within each scenario considered here. Then we compared the best model of each scenario to the best models from other scenarios. In addition, to test the robustness of our conclusions, we also compared all models for which the likelihood of observed data fell well within the distribution of simulated data in a single model selection procedure. Moreover, and for the same reason, we applied an alternative nesting strategy where we nested migration dynamics within population size dynamics. To estimate the power of our procedure to distinguish between selected models we generated 1,000 pseudo-observed datasets for each model and checked how many times the ABC model choice procedure failed to correctly predict the true model [41]. Each pseudo-observed dataset produced by a considered model (the true model in this case) was treated as observed data and used to calculate marginal densities of all compared models. Bayes factors were used to judge if a selected model coincided with the true model. Our demographic model evaluation procedure included slight adjustments of prior ranges for particular model parameters and this adjustment procedure may influence model selection by favouring more optimized models over less optimized once. This is the consequence of Bayes factor calculations that are based on the marginal likelihoods of the models considered: the marginal likelihood of a model will be higher if the selected prior probability distributions are more similar to the true posterior probability distributions. Thus, to validate our model choice analyses we ran additional simulations to evaluate the sensitivity of the model posterior probability distributions to choices of different prior distribution. The best model (RMASC) was run with 4 different ‘sub-optimal’ prior ranges (the ranges of sub-optimal priors corresponded to the adjustment we made during exploratory simulations, Table S4). For each sub-optimal model we followed the same hierarchical model choice procedure as for our original simulations.

### Validation of the Estimation Procedure

We validated the chosen estimation procedure and summary statistics by checking for a potential bias in the posterior distributions [18], [42]. We generated 1,000 pseudo-observed datasets with known parameter values and computed coverage property of the posterior distributions obtained with ABC-GLM regression adjustment. The uniformity of the posterior quantiles for each parameter was checked with a Kolomogorov-Smirnov test and its significance was obtained after Bonferroni correction. To verify if retained simulations were exploring the appropriate space of summary statistics, we plotted PLS components together with observed transformed statistics. To check the power to estimate individual parameters we computed the coefficient of variation (R^2^) by regressing PLS components against model parameters [79]. In addition, we computed the root mean squared error of the mode (RMSE) for each parameter to check the accuracy of the mode as a point estimate [42].

All simulations were run on linux clusters at Uppsala Multidisciplinary Center for Advanced Computational Science (UPPMAX). Often we run several hundred simulations in parallel and we used in-house scripts to generate random seed numbers for each simulation to avoid the risk of several simulations being identical.

### PSMC Analysis

Changes in effective population size over time were assessed by pairwise sequentially Markovian coalescent model analysis [15]. The model estimates the local time to the most recent common ancestor based on a single whole-genome diploid sequence and uses information from the rates of the coalescent events in a given epoch to infer N_e_ at a given time [15], [99]. Since the method heavily relies on the distribution of polymorphic sites across the genome, it can only be used when both alleles are called with high confidence (i.e., when per-site coverage is high). Thus, we used the diploid sequence of the male collared flycatcher sequenced for genome assembly (mean coverage 85×; [57]). Data was filtered by excluding sites at which read depth was more than twice or less than half of the average read depth, the root mean squared mapping quality of reads covering the site was below 25, the site was within 10 bp around predicted indels and the inferred consensus quality was below 20. A generation time of 1 year and a mutation rate of 1.4×10^−9^ year/site were applied (based on our ABC analysis). The settings of the PSMC analysis (-p and –t options) were chosen manually according to suggestions given by Li and Durbin ([15], https://github.com/lh3/psmc). To check for variance in N_e_ estimates we performed a total of 100 bootstrap tests.

## Supporting Information

Figure S1 Density distribution of the PLS components of retained simulations (black circles) and observed data (red dot).
(TIF)

Figure S2 Posterior quantile distributions for RMASC model parameters.
(TIF)

Figure S3 Posterior quantile distributions for RUMASC model parameters.
(TIF)

Table S1 Scenarios and models investigated in the study.
(DOCX)

Table S2 Prior ranges for ‘sub-optimal’ RMASC model.
(DOCX)

Table S3 Prior and posterior distributions of recent unidirectional migration and ancient size change (RUMASC).
(DOCX)

Table S4 Model choice with ‘not-optimized’ simulations.
(DOCX)
