# Supplementary figures and images for: Demographic Divergence History of Pied Flycatcher and Collared Flycatcher Inferred from Whole-Genome Re-sequencing Data
Source: PLoS Genet. 2013 Nov 7;9(11):e1003942. doi: 10.1371/journal.pgen.1003942 (PMC3820794; doi:10.1371/journal.pgen.1003942)

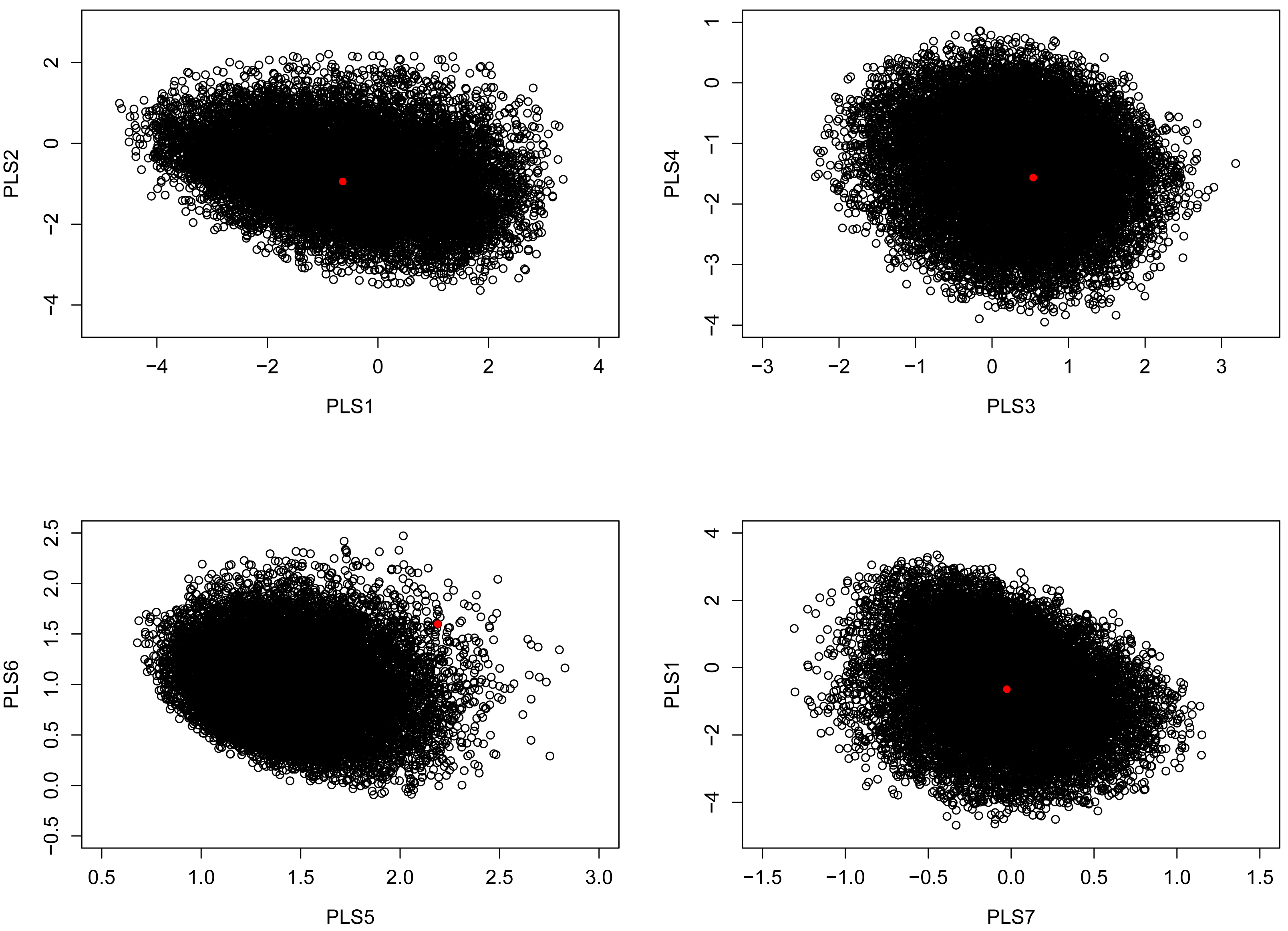

Supplement: Figure S1 — Density distribution of the PLS components of retained simulations (black circles) and observed data (red dot). (TIF) [file pgen.1003942.s001.tif]

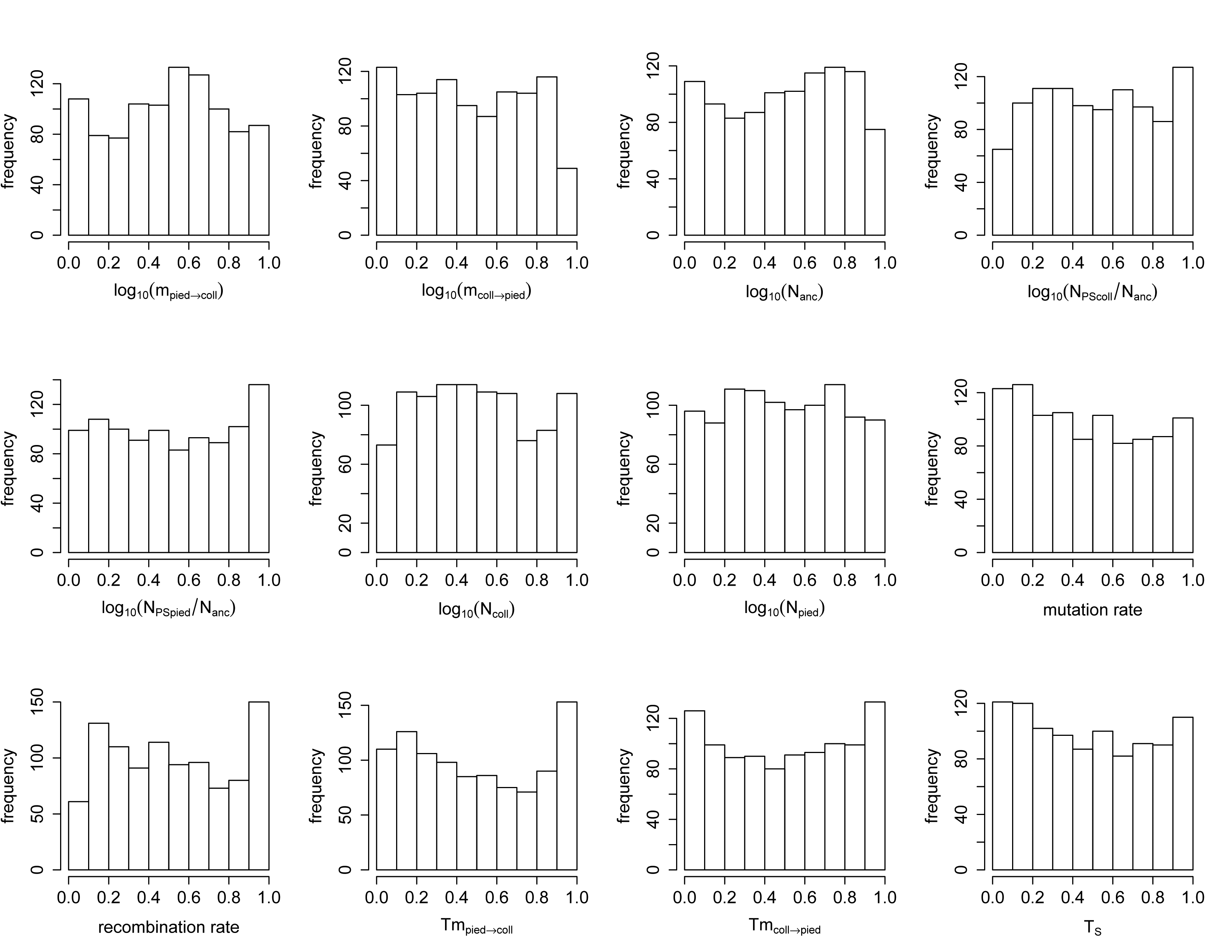

Supplement: Figure S2 — Posterior quantile distributions for RMASC model parameters. (TIF) [file pgen.1003942.s002.tif]

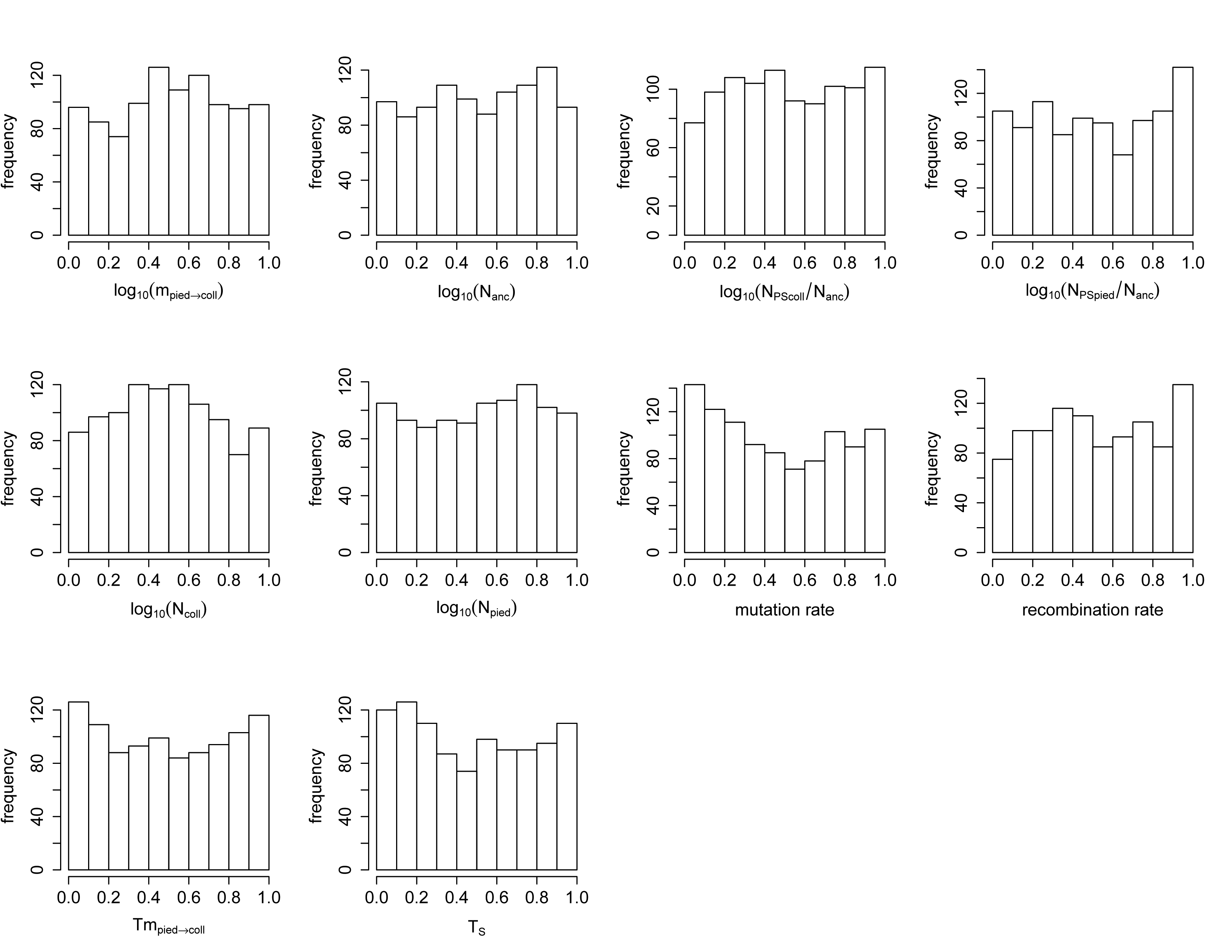

Supplement: Figure S3 — Posterior quantile distributions for RUMASC model parameters. (TIF) [file pgen.1003942.s003.tif]
