# Supplementary material for: Demographic Divergence History of Pied Flycatcher and Collared Flycatcher Inferred from Whole-Genome Re-sequencing Data
Source: PLoS Genet. 2013 Nov 7;9(11):e1003942. doi: 10.1371/journal.pgen.1003942 (PMC3820794; doi:10.1371/journal.pgen.1003942)
Supplement: Table S1 — Scenarios and models investigated in the study. (DOCX) [file pgen.1003942.s004.docx]

Table S1. Scenarios and models investigated in the study.

| Scenario | Model | PP | Power |
| --- | --- | --- | --- |
| Isolation | ICS |  |  |
|  | IRSC |  |  |
|  | **IASC** |  |  |
|  |  |  |  |
| Constant migration | **CMCS** | 0.99 | 0.91 |
|  | **CMRSC** | 0.01 | 0.85 |
|  | CMASC |  |  |
|  |  |  |  |
|  |  |  |  |
| Recent migration | **RMCS** | 0.01 | 0.53 |
|  | **RMRSC** | 0.00 | 0.68 |
|  | **RMASC** | 0.99 | 0.73 |
|  |  |  |  |
| Ancient migration | AMCS |  |  |
|  | AMRSC |  |  |
|  | AMASC |  |  |
|  |  |  |  |
| Recent and ancient migration | **RAMCS** | 0.02 | 0.87 |
|  | **RAMRSC** | 0.98 | 0.82 |
|  | RAMASC |  |  |

PP - posterior probabilities of the models for which the likelihood of observed data fell within the distribution of simulated data (bold). Power – a power of model choice procedure to distinguish between compared models. The value represents a fraction of properly predicted models computed based on 1000 pseudo-observed dataset. Model abbreviations as described in the main text and in Figure 2.
