# Supplementary material for: Demographic Divergence History of Pied Flycatcher and Collared Flycatcher Inferred from Whole-Genome Re-sequencing Data
Source: PLoS Genet. 2013 Nov 7;9(11):e1003942. doi: 10.1371/journal.pgen.1003942 (PMC3820794; doi:10.1371/journal.pgen.1003942)
Supplement: Table S2 — Prior ranges for ‘sub-optimal’ RMASC model. (DOCX) [file pgen.1003942.s005.docx]

Table S2. Prior ranges for ‘sub-optimal’ RMASC model.

|  | Not-optimized priors | | | | | | | |
| --- | --- | --- | --- | --- | --- | --- | --- | --- |
|  | Simulation 1 | | Simulation 2 | | Simulation 3 | | Simulation 4 | |
| Parameter | minimum | maximum | minimum | maximum | minimum | maximum | minimum | maximum |
| log_10_(N_coll_) | 4.5 | 6 | 4.5 | 6 | 4.5 | 6 | 4.5 | 6 |
| log_10_(N_pied_) | 4.4 | 5.2 | 4.4 | 5.2 | **4.4** | **6** | **4.4** | **6** |
| log_10_(N_anc_) | 4.5 | 6 | 4.5 | 6 | 4.5 | 6 | 4.5 | 6 |
| log_10_(N_PScoll_/N_coll_) | -3 | 3 | -3 | 3 | -3 | 3 | -3 | 3 |
| log_10_(N_PSpied_/N_pied_) | -1 | 3.5 | **-3** | **3** | -1 | 3.5 | **-3** | **3** |
| log_10_(M_pied->coll_) | **-3** | **0.6** | -1.5 | 0.6 | -1.5 | 0.6 | **-3** | **0.6** |
| log_10_(M_coll->pied_) | **-6** | **0.6** | -4 | 0 | -4 | 0 | **-6** | **0.6** |
| Tm_pied->coll_ | 150 | 25000 | 150 | 25000 | 150 | 25000 | 150 | 25000 |
| Tm_coll->pied_ | 150 | 25000 | 150 | 25000 | 150 | 25000 | 150 | 25000 |
| T_s_ | 10000 | 1000000 | 10000 | 1000000 | 10000 | 1000000 | 10000 | 1000000 |
| μ x10^-9^ | 1 | 5 | 1 | 5 | 1 | 5 | 1 | 5 |
| *r* x10^-8^ | 0.1 | 10 | 0.1 | 10 | 0.1 | 10 | 0.1 | 10 |

Bold values indicate prior ranges different from the original best model.
