# Supplementary material for: Demographic Divergence History of Pied Flycatcher and Collared Flycatcher Inferred from Whole-Genome Re-sequencing Data
Source: PLoS Genet. 2013 Nov 7;9(11):e1003942. doi: 10.1371/journal.pgen.1003942 (PMC3820794; doi:10.1371/journal.pgen.1003942)
Supplement: Table S3 — Prior and posterior distributions of recent unidirectional migration and ancient size change (RUMASC). (DOCX) [file pgen.1003942.s006.docx]

Table S3. Prior and posterior distributions of recent unidirectional migration and ancient size change (RUMASC).

|  | Prior^a^ | | Estimation validation | | |  | Posterior characteristics | | | | | |
| --- | --- | --- | --- | --- | --- | --- | --- | --- | --- | --- | --- | --- |
|  |  |  |  |  |  |  | HPDI 50 | | HPDI 90 | | HPDI 95 | |
| Parameter | minimum | maximum | P value^b^ | R^2 c^ | RMSE^d^ | Mode | Lower | Upper | Lower | Upper | Lower | Upper |
| log_10_(N_coll_) | 4.5 | 6 | 0.005 | 0.57 | 0.29 | 4.81 | 4.68 | 5.00 | 4.52 | 5.26 | 4.51 | 5.37 |
| log_10_(N_pied_) | 4.2 | 5.2 | 0.164 | 0.59 | 0.23 | 4.37 | 4.28 | 4.49 | 4.21 | 4.68 | 4.20 | 4.75 |
| log_10_(N_anc_) | 4.5 | 6 | 0.253 | 0.45 | 0.26 | 5.74 | 5.59 | 5.85 | 5.39 | 6.00 | 5.29 | 6.00 |
| log_10_(N_PScoll_/N_coll_) | -3 | 3 | 0.263 | 0.31 | 1.51 | 2.31 | 1.61 | 2.8 | 0.29 | 2.98 | -0.18 | 2.98 |
| log_10_(N_PSpied_/N_pied_) | -1 | 3.5 | 0.007 | 0.46 | 0.84 | 3.03 | 2.58 | 3.4 | 1.53 | 3.49 | 1.16 | 3.49 |
| log_10_(M_pied->coll_)^e^ | -1.5 | 0.6 | 0.011 | 0.49 | 0.7 | -1.01 | -1.27 | -0.67 | -1.48 | -0.15 | -1.48 | 0.03 |
| Tm_pied->coll_ | 150 | 0.6 | 0.085 | 0.12 | 0.15 | 10343 | 5491 | 16185 | 1657 | 21734 | 939 | 22646 |
| T_s_ | 10000 | 1000000 | **0.002** | 0.25 | 5.7 | 226634 | 147488 | 321319 | 102262 | 524476 | 102262 | 614488 |
| μ x10^-9^ | 1 | 5 | **0** | 0.43 | 0.83 | 1.48 | 1.15 | 1.91 | 1.01 | 2.8 | 1.01 | 3.13 |
| *r* x10^-8^ | 0.1 | 10 | 0.133 | 0.1 | 2.02 | 6.57 | 4.44 | 8.18 | 2.16 | 9.73 | 1.65 | 9.98 |

^a^ all priors are uniformly distributed

^b^ P value computed with Kolmogorov-Smirnoff test; bold values indicate significant deviations from uniformity after Bonferroni correction

^c^ coefficient of determination

^d^ average root mean square error

^e^ M_pied->coll_ equals 4N_0_m_pied->coll_ ; N_0_ = 10,000
