# Supplementary material for: Demographic Divergence History of Pied Flycatcher and Collared Flycatcher Inferred from Whole-Genome Re-sequencing Data
Source: PLoS Genet. 2013 Nov 7;9(11):e1003942. doi: 10.1371/journal.pgen.1003942 (PMC3820794; doi:10.1371/journal.pgen.1003942)
Supplement: Table S4 — Model choice with ‘not-optimized’ simulations. (DOCX) [file pgen.1003942.s007.docx]

Table S4. Model choice with ‘not-optimized’ simulations.

|  |  | Simulation 1 | Simulation 2 | Simulation 3 | Simulation 4 |
| --- | --- | --- | --- | --- | --- |
| Recent migration models | Model | PP | PP | PP | PP |
|  | **RMCS** | 0.01 | 0.02 | 0.02 | 0.04 |
|  | **RMRSC** | 0.01 | 0.01 | 0.01 | 0.02 |
|  | **RMASC** | 0.98 | 0.97 | 0.97 | 0.94 |
|  |  |  |  |  |  |
| Best models from each scenario | **IASC** | 0.06 | 0.11 | 0.07 | 0.10 |
|  | **CMCS** | 0.06 | 0.11 | 0.07 | 0.10 |
|  | **RMASC** | 0.88 | 0.77 | 0.84 | 0.80 |
|  | **RAMRSC** | 0.00 | 0.00 | 0.00 | 0.00 |

PP - Posterior Probability
